# Blending Cognitive Analytic Therapy With a Digital Support Tool: Mixed Methods Study Involving a User-Centered Design of a Prototype App

**DOI:** 10.2196/20213

**Published:** 2021-02-01

**Authors:** Katherine Easton, Stephen Kellett, Martin Cooper, Abigail Millings, Jo Varela, Glenys Parry

**Affiliations:** 1 School of Education University of Sheffield Sheffield United Kingdom; 2 Sheffield Health & Social Care NHS Foundation Trust University of Sheffield Sheffield United Kingdom; 3 Software Engineering, Graphics and Multimedia Sheffield Hallam University Sheffield United Kingdom; 4 Centre for Behavioural Science and Applied Psychology Sheffield Hallam University Sheffield United Kingdom; 5 Derbyshire Community Health Service NHS Foundation Trust Derbyshire United Kingdom; 6 Catalyse UK Sheffield United Kingdom

**Keywords:** cognitive analytic therapy, digital app, relational awareness, user-centered design, acceptability, user testing

## Abstract

**Background:**

Patients can struggle to make good use of psychotherapy owing to deficits in awareness, and digital technologies that support awareness are at a premium. Currently, when patients participate in cognitive analytic therapy (CAT), the technology supporting relational awareness work involves completion of paper-based worksheets as between-session tasks.

**Objective:**

We aimed to design, with therapists and patients, a prototype digital mobile app. This was to help patients better engage in the “recognition” phase of the CAT treatment model by providing an unobtrusive means for practicing relational awareness with dynamic feedback on progress.

**Methods:**

A national online survey was conducted with CAT therapists (n=50) to determine readiness for adoption of a mobile app in clinical practice and to identify core content, functionality, and potential barriers to adoption. A prototype mobile app based on data and existing paper-based worksheets was built. Initial face-to-face user testing of the prototype system was completed with three therapists and three CAT expatients.

**Results:**

Among the therapists surveyed, 72% (36/50) reported not currently using any digital tools during CAT. However, the potential value of a mobile app to support patient awareness was widely endorsed. Areas of therapist concern were data security, data governance, and equality of access. These concerns were mirrored during subsequent user testing by CAT therapists. Expatients generated additional user specifications on the design, functionality, and usability of the app. Results from both streams were integrated to produce five key changes for the reiteration of the app.

**Conclusions:**

The user-centered design process has enabled a prototype CAT-App to be developed to enhance the relational awareness work of CAT. This means that patients can now practice relational awareness in a much more unobtrusive manner and with ongoing dynamic feedback of progress. Testing the acceptability and feasibility of this technological innovation in clinical practice is the next stage in the research process, which has since been conducted and has been submitted. The important challenges of data protection and governance must be navigated in order to ensure implementation and adoption if the CAT-App is found to be acceptable and clinically effective.

## Introduction

### Cognitive Analytic Therapy

Cognitive analytic therapy (CAT) is a brief, relational, and integrative psychotherapy most often offered in secondary care services in 16- or 24-session formats to patients with complex psychological needs. It can also be delivered in an eight-session version for less complex problems in primary care [1]. Rather than relying on a treatment protocol that therapists follow on a session-by-session basis, CAT has been described as a “humanized and skilled” psychotherapy [2], requiring a relatively high level of training and supervision to practice competently. The competences for CAT delivery are well-specified [3,4], where CAT therapists implement a three-phase approach in terms of reformulation, recognition, and revision. However, within this framework, therapists exercise judgement, draw on a range of techniques, and adapt the delivery and the methods used to be responsive to the needs and limitations of each patient [5].

The reformulation phase of CAT is supported through early production of narrative and diagrammatic reformulations based on the procedural sequence object relation model [6]. The therapist and client negotiate a shared understanding of target problems and the target problem procedures. These target problem procedures are ways in which the client relates to himself or herself (self-to-self reciprocal roles and associated procedural sequences) and to others (self-to-other and other-to-self reciprocal roles and associated procedural sequences), including the therapist. Crucially, roles and sequences are linked through narrative and diagrammatic reformulations to early developmental traumatic or neglectful experiences and their repetition in adult life through the action of procedural memory [7]. Once the reformulation is agreed, the recognition phase helps the client become aware of the ways these patterns operate in everyday life. The recognition phase tries therefore to build relational awareness of the manner in which the patient relates to himself or herself and others (and vice versa), including the therapist. The revision phase of CAT is the “change work” phase of the model and supports patients in changing roles and sequences, working within the individual’s zone of proximal development [5].

The recognition phase of CAT is therefore an integral part of the change process. Because reciprocal roles and associated procedures are enacted automatically, revision (ie, change) requires new awareness of them. CAT therapists therefore try to support patients in building better relational awareness of when they are about to enact, are currently enacting, or have just enacted a problematic role or procedure with themselves or others. The main aim of the recognition phase of CAT is that the patient builds awareness through being able to occupy the “observing eye” position. Indeed, an observing eye is often drawn on sequential diagrammatic reformulations in order to remind patients to occupy this position. This position has much in common with the “self-as-context” process of acceptance and commitment therapy [8]. There is evidence that outcome in CAT is heavily dependent on the patient being able to occupy the “observing eye” position, as from this position of awareness, change is then possible [9].

Currently, the self-monitoring tools that support within- and between-session relational awareness practices are wholly paper based. These consist of two types of worksheets. The first between-session tool enables the patient to make observations in daily life of key interpersonal events and resulting thoughts, emotions, and actions, which link to their problem procedures. This written diary record is brought to the session for review and analysis. The second within-session tool lists the patient’s target problems and associated target problem procedures in a standardized format. The patient rates the manner in which awareness is improving, static, or deteriorating and whether revision is improving, static, or deteriorating. In clinical practice, however, patients have repeatedly reported to the authors (SK, GP, and JV) that diary keeping of this very personal kind is inconvenient, physically clunky, or potentially embarrassing, especially when observed by others. The development and use of a digital tool suitable for a smartphone or tablet could provide privacy, ease, and convenience, so that patients can practice relational awareness.

### Digital Technology and Mental Health

The value of digital technology to increase access, availability, and innovation in mental health care is high on national and global research and policy agendas [10-14]. Digital tools, such as mobile phones, tablets, laptops, and wearable devices, are ubiquitous in modern life. For some patients, the greater convenience, accessibility, and availability afforded by digital health technology have been found to outweigh the advantages of face-to-face contact with health professionals [15]. However, evidence suggests that for digital mental health to be as effective as traditional face-to-face delivery of care, a blended/augmented or guided approach with some real-time personal support is preferable to using digital tools in isolation from human contact [16]. The value of such a blended approach was highlighted in a recent James Lind Alliance Priority Setting exercise on digital mental health by patients and clinicians [11]. Mobile technologies, such as smartphones and mobile apps, have been suggested to be superior treatment delivery platforms, because they allow constant access to treatment materials [17].

### CAT and Digital Support

Currently, there are no digital apps that are influenced by or support the practice of CAT. CAT principles have been applied to a six-session guided self-help intervention, which has been feasibility tested for use in the United Kingdom Increasing Access to Psychological Therapies (IAPT) services with good outcomes [18]. This demonstrates that it is possible to translate the model into other easily accessible formats. While it may be possible to develop a computer-based self-help program based on CAT principles, the therapy itself is intrinsically personally delivered, since the relationship between the therapist and the patient is itself a central focus of procedural recognition and revision. The purpose of this project was therefore different. We aimed to support blended patient engagement in the recognition phase of CAT, rather than develop computerized delivery of CAT.

There are clearly identifiable aspects of the model that could be better supported through the development of a mobile app, such as easy access to narrative and sequential diagrammatic reformulations; monitoring of mood, target problems, and target problem procedure methods for logging and evaluating the effectiveness of a change strategy; and storing the “goodbye letters” from both patients and therapists that are a distinctive aspect of how endings are negotiated with the model. There are therefore potential benefits of an app to augment therapy by making the process more accessible both between sessions or after the therapy has finished, by increasing the ways in which a patient can hold the therapy in mind outside the therapy room (ie, an internalization process) [19] and make use of the treatment model [20]. There is evidence of reduced interpersonal problems during the follow-up from CAT [21], and therefore, an app may be particularly important in terms of supporting the durability of relational change through ongoing relational awareness.

The notion of taking a relational awareness approach is novel in terms of digital health care, as the plethora of cognitive-behavioral therapy (CBT) apps tend to focus on symptom monitoring, and associated thinking and behavior change [17]. Unlike many “off-the-shelf” CBT or therapy support apps, it is essential that any CAT app be customized to each individual user owing to the often highly idiosyncratic and person-specific approach of the model [5]. The content of the app should therefore be created through collaboration between both the therapist and patient and should mirror the content of the sessions and the phased approach of CAT. To summarize, the aim of this research was to design and develop a prototype digital app to support patients accessing CAT, particularly to support the out-of-session relational awareness tasks of the recognition phase of the therapy.

## Methods

### Design

A user-centered design approach was adopted as this was in line with the Medical Research Council (MRC) guidelines [22], suggesting process evaluation and feasibility testing before moving onto effectiveness testing. A working group of CAT experts, psychologists, and an app developer collaborated to conduct a mixed methods study with the following stages: stage 1, collaborative design of the core clinical content of the system in order to develop an initial prototype app (online questionnaire survey for therapists); stage 2, scoping out functional and nonfunctional/operational requirements for the system, including interface features and visual appearance, and addressing issues of acceptability and usability (CAT therapist and expatient user testing); stage 3, technical development and refinement, making it ready for feasibility testing (technical development).

### Stage 1: Clinical Content

In stage 1, a bespoke questionnaire survey was developed and conducted in Qualtrics (Qualtrics). The sample for the online survey was recruited via an advertisement in “Reformulation,” the house journal of the Association for Cognitive Analytic Therapy (ACAT). Any CAT therapist reading the journal could access the online survey via a link provided. The survey ran from September 20, 2016, to October 20, 2016. Information sheets and informed consent forms were included at the start of the online survey. The survey contained a mix of closed and open items relating to current eHealth usage in practice, components of CAT that should form an aspect of app content, and suggestions for names for the app. Closed items asked respondents to identify the importance of suggested clinical content in the app on a 5-point Likert scale, ranging from “not at all important” (1) to “very important” (5). Open items asked for suggestions for key features and allowed respondents to share concerns and considerations for a digital CAT tool. The clinical content items were initially developed in a consensus meeting with CAT therapists on the team (SK, GP, and JV). Through the survey, we could identify which contents a larger sample of therapists felt were vital to ensure theoretical fidelity of the intervention.

### Stage 2: User Testing

In stage 2, informal one-to-one user testing was used with co-operative evaluation methods. Specifically, a cognitive walk-through method was used where users verbalize their thinking as they navigate the app [23] in order to gather data for the design of the prototype app. Co-operative evaluation methods attempt to get users to see themselves as “collaborators” in the development of health technology and any associated evaluation. The aim of this was to identify ways to make the app more intuitive for future users. The cognitive walk through allows the researchers/designers to assess the usability of the system and assign causes to usability problems early in the design process. We chose one-to-one testing over focus group methods, as clients may wish to use their own experiences and symptoms to work through the app in testing and such discussions may not arise in a group setting. CAT therapists and expatients for the user testing were recruited through an organization that provides CAT in the independent sector on a not-for-profit basis (Catalyse Sheffield Psychotherapy Practice). Eligible therapist participants were required to have completed CAT practitioner (2-year training) or CAT psychotherapy (4-year training) training and have a current caseload of CAT patients. Eligible service user participants were required to have recently undertaken and completed a full course of one-to-one 16- or 24-session CAT, regardless of the outcome. Patients who recently completed the course were selected as opposed to active patients for the following two reasons: (1) not interfere with the therapeutic process with this early stage research and (2) have the full experience of CAT where the end stages are particularly salient for procedural revision. Patient participants were required to be nominated by therapists in order for them to be selected for recruitment. The research was advertised internally to therapists via email, and therapists were asked to identify any former clients who they felt might be suitable for the research. Potential patient participants were contacted initially by the therapist and were asked to email the researcher (KE) to express interest and receive an information sheet and consent form via email. We had no participant numbers in mind, and we aimed to recruit a small sample of both users in order to explore a range of issues and identify any consensus problems with the first prototype version.

User testing occurred face to face either at The University of Sheffield or at the Catalyse Sheffield offices. During user testing, the participants (therapists and expatients) were shown a prototype version of the app and asked to work through the stages and processes of the app. During user testing, participants were encouraged to “think aloud” during their engagement with the app, so that thoughts and feelings could be recorded (ie, cognitive walk though). The testing lasted for as long as was needed to work through the app; however, we estimated that this would take no longer than an hour based on our previous user testing. The user testing was audio recorded in order to track how users navigated the features of the app and the internal commentary that occurred. When service users worked through the app and entered data relating to the nature of their condition, they were reminded that all data were confidentially stored and destroyed once the testing was complete. They were also free to use hypothetical problems rather than their own, and these were provided if preferred. User testing of the basic prototype app (based on findings from the questionnaire research) began in January 2017.

### Stage 3: Technical Development

The following two prototype apps were created: a therapist web application and a patient mobile app. The therapist application is a web-based tool allowing therapists to create profiles for patients by updating information about the client’s target problems and target problem procedures. This can potentially include images and videos that the client may find useful in their therapy. Once a patient is set up on this system, the therapist’s web application will allocate a personal identification number (PIN) to allow the patient to access the mobile app. The current web application is hosted on a Sheffield Hallam University server. Data are stored on a Google Firebase Cloud service [24]. Data are fully anonymized via the PIN process.

The mobile app is designed for patients to use on their mobile phone or tablet as an “app.” The app is accessed via a PIN allocated through the set-up procedure undertaken by the therapist in the web application outlined above. Once patients have access to the app, they can review and rate the current recognition and revision (0-100 scale from ineffective recognition/revision to effective recognition/revision) of their personalized target problems and target problem procedures, see their personal narrative and diagrammatic personal reformulations, and provide a current mood rating. Patients rated their mood on a slider scale (0-10 points) in terms of sad/happy, anxious/calm, and excited/bored (reverse scored). Mood items reflected the pleasure (valence) and arousal (activation) dimensions of the core affect [25]. Feedback on current and all previous attempts at the recognition and revision of target problems, target problem procedures, and mood were presented as progress graphs. Therefore, the app contains a combination of ideographic content that is highly personalized and a nomothetic measurement for standardized clinical outcome measurement and research purposes.

These data are stored anonymously in the same Google Firebase Cloud accessed by the therapist web application.

The patient app has been accepted as an app on both the Apple iOS and Google Android App stores. Both versions of the app were built with the Ionic hybrid framework [26].

### Data Analysis

Data from the stage 1 therapist survey were extracted, and descriptive statistics were calculated to identify the most important clinical features of the app. Any clinical features that were reported as “extremely important” or “very important” by over 50% of the sample were included in the prototype. Audio recordings from the stage 2 user testing conducted by KE were transcribed, and a thematic analysis was conducted by KE to identify key user requirements to feed into the app development. Analysis was not iterative but summative. Data saturation was not sought. We were looking to identify primary areas of concern with respect to functionality and usability in a short span of time to improve the next iteration of the app for follow-on feasibility testing. All themes were brought to a consensus team meeting along with transcriptions for discussion and validation across the team.

Ethical approval for the research was obtained from The University of Sheffield (no: 011086 and 011553).

## Results

### Stage 1: Clinical Content

Sixty-four CAT therapists accessed the survey, of which 62 provided consent to participate. Twelve did not complete the survey with consent, leaving a final sample size of 50 participants. The majority (n=36, 72%) of respondents reported not using any eHealth approaches in their current clinical practice. The 14 (28%) respondents who did use some eHealth support reported integrating a range of apps for different purposes. The most commonly recommended apps were those that support mindfulness and meditation, such as Headspace (n=6), Moodnotes (n=1), Moodshift (n=1), Buddify (n=1), and those that encourage connections with others/peer support, such as Big White Wall (n=1) and Silent Secret (n=1). Only one respondent mentioned recommending an app to monitor mood via the Patient Health Questionnaire (PHQ)-9 [27]. Although one respondent did not use any apps during CAT therapy, the respondent mentioned the following:

…several of my private patients use their phones for diary keeping which they find helpful. Likewise, they take pictures of maps to take with them and look at in between sessions. I often use my iPad camera and phone, to snap maps sketched out on a white board which I can then either transfer onto paper or print out and delete. I have noticed there is a sense of being able to own a piece of the fluid experience within the session.

Table 1 presents the features that were felt to be most important in a potential app to support CAT. The following features were rated as extremely important: “A place to store the *map* (ie, the sequential diagrammatic reformulation), so it can be accessed by clients on their phone” (21/50, 42%) and “A list of personal exits developed in the therapy” (22/50, 44%). Moreover, the following were rated as very important: “Ability to write a short reminder/diary of events (ie, enactments, etc) during the week, so they can be better discussed during therapy sessions;” “Rating for recognition of target problem procedures;” “Rating of revision of target problem procedures;” and “Rating of the severity of the person’s target problems” (Table 1).

**Table 1:** Clinical features for inclusion in the app.

Item	Importance (N=50), n (%)				
	Not at all important	Slightly important	Moderately important	Very important	Extremely important
A list of personal exits developed in the therapy	1 (2%)	1 (2%)	7 (14%)	19 (38%)	22 (44%)
A place to store the *map*, so it can be accessed by clients on their phone	2 (4%)	4 (8%)	6 (12%)	17 (34%)	21 (42%)
Ability to write a short reminder/diary of events (ie, enactments, etc) during the week, so they can be better discussed during therapy sessions	2 (4%)	0 (0%)	12 (24%)	23 (46%)	13 (26%)
Rating for recognition of target problem procedures	1 (2%)	6 (12%)	12 (24%)	21 (42%)	10 (20%)
Rating of revision of target problem procedures	1 (2%)	7 (14%)	12 (24%)	20 (40%)	10 (20%)
A place to store the goodbye letter, so that it can be accessed by clients on their phone	3 (6%)	10 (20%)	12 (24%)	18 (36%)	7 (14%)
A place to store the reformulation letter, so that it can be accessed by clients on their phone	3 (6%)	9 (18%)	16 (32%)	15 (30%)	7 (14%)
Rating of the severity of the person’s target problems	2 (4%)	13 (26%)	16 (32%)	14 (28%)	5 (10%)
Session list/countdown to total number of sessions attended	3 (6%)	6 (12%)	22 (44%)	16 (32%)	3 (6%)
Alarms and reminders for therapy	3 (6%)	9 (18%)	24 (48%)	10 (20%)	4 (8%)

When asked what other *features* should be included other than those listed in Table 1, six CAT therapists indicated that an interactive version of the sequential diagrammatic reformulation (ie, the *map* of patient’s reciprocal roles, self-states, and procedures) could be useful for patients to track their progress and update with exits in real time as they progress. In addition, six CAT therapists recommended the use of reminders, prompts, or alarms that patients could set for themselves on the app to remind them to complete the between-session recognition work. The option to use other creative visual tools in the app to support progress and change was highlighted by three CAT therapists. A glossary of key terms used during CAT was highlighted as potentially useful, in addition to links to crisis support, additional CAT resources, and literature. A storage area for recording personalized “exits” (ie, new ways of relating to others or self) being useful in supporting and implementing successful change strategies was noted by two CAT therapists.

These recommendations were logged but not incorporated into the prototype for initial design work, as they were not integral to the therapeutic process and the inclusion of an interactive map, for instance, represents a larger technical challenge than is needed in this initial design phase (data available on request to the corresponding author).

Respondents were asked if there were any other *issues for consideration* when developing digital technology to support CAT, and the issues of data protection and confidentiality were crucial and key concerns for all respondents in the survey. The need for the app to be accessible to all was another core requirement identified in the questionnaire feedback (n=9), in relation to cost, ease of use, and accessibility for clients with learning disabilities and visual impairments.

### Stage 2: User Testing

The two applications (therapist web application and patient mobile app) were user tested with a small sample of three therapists. All therapists were female. Two were clinical psychologists, and one was a counselling psychologist. All were experienced CAT therapists and supervisors. Three expatients took part in the testing. Two were female, and one was male. The CAT therapists viewed both applications (ID1, self-reported novice; ID2, expert; ID3, proficient user of technology), and expatients used the patient app (all self-reported proficient users of technology), working through it on their own and thinking aloud as they did so for between 30 and 60 minutes.

#### Therapist Feedback

Two main themes emerged during therapist user testing, including data protection and fidelity to the intervention process. Two therapists stressed the need for clear and early information on data protection and data sharing for patients on first viewing of the patient app. The following comments were made:

This needs to feature a pretherapy agreement on first use of the app (or something similar) for data protection and storage information. You wouldn’t need to access this again.

ID3

I had assumed this would have a high level of security. You need to think carefully about this. We need to know where the data are being stored and need to have an agreement around it between the app provider, therapist, organization, and client. Does the app provider host the data? Does the clinical organization have responsibility for the data? Clients will need to know who is looking after their data and what is happening to it.

ID2

You will need a statement on the app which refers to the following: I keep records in accordance with my professional body’s guidelines and the data protection legislation. These records are kept confidentially and securely, with a personal record number. Records are kept for 7 years.

ID3

The dangers of not fully considering data protection were highlighted. One therapist made the following comment:

I used (NAMED APP) with a client, and after it shut down, she had no idea where all the data are now.

ID2

When testing the therapist web application, the need for it to be secure and separate to any client access was stressed as important for data protection and privacy.

There needs to be separate areas, one for administrative tasks for the therapist and one collaborative work space for the client that the therapist can also access. It's important to be able to access clients via a home page search function and drop down menu option – this function should be password protected, and if in the same room as a client, they should only be able to see their data – not a list of people – even if nicknames are used.

ID3

All therapists interviewed stated that they felt the app should more accurately reflect the way in which target problem procedures are developed and shared with patients. They commented as follows:

I found it easy to use but it needs to reflect TPP.

ID1

It needs to reflect TPP. It’s too prescriptive and doesn’t allow for individualized ways of working. I got frustrated very quickly and not happy with the layout. Too complicated. When working with a client a lot of work is sketchy to begin with – create a map and refine as sessions progress. This app allows for one map. You would need to be able to upload newer versions as you progress and archive older ones.

ID2

I wondered why it doesn’t reflect TPP. You need to work from CAT documents and build on it. If the app does less than what you can access face to face and on paper, then no one will use it. You need to have target – pattern – leading onto recognition, revisions, and progress toward the target problem listed (for clients, and as a consequence, the therapist).

ID3

With respect to the patient app, all three therapists felt the mood tracker function should be personalized. They commented as follows:

Patients need to be able to personalize the emotions within this. This part needs more work. Patients need more options.

ID1

This needs to be more creative. Photos or emoji’s, that would make it easier for people with learning difficulties and younger users.

ID2

This needs to more creative and personalized, but we need to be able to aggregate data! You can’t aggregate photos, for example, so it still needs to be temporal scale of some kind.

ID3

In this prototype, the “exit” list for clients to refer to was labeled “to do list,” which caused confusion. The following comments were made:

I like this function, an area to keep working on, this needs to be a flexible space where patients can add resources as they go and it should link to the map.

ID1

To do lists? What’s this exits? I like this. Could it link to a bank of tools that clients draw on?

ID2

All therapists felt that a digital support tool was a good idea. However, therapist two raised a concern that the app felt a little paternalistic and, as it stood, represented more of a research tool than a therapy app. Therapist three also emphasized considering patients’ expectations when they use it and mirrored therapist two’s thoughts on clarifying why data are being held and what will be done with the data.

#### Expatient Feedback

The themes identified by past patients were (1) usability and personalization, (2) lack of concern with privacy, and (3) convenience of a digital tool.

None of the users found the flow of content intuitive. All three suggested a drop down menu at the top of the app, so that they would not need to backtrack through stages. The following comments were made:

This (the navigation system) needs to be at the top left like most other apps. I don’t want to click back each time to get out of targets.

ID1

You need to make it easier to move about the app. It’s a bit clunky at the moment, moving about though the sections. It needs to flow better.

ID2

The design and colors of the app were not thought to be particularly appealing; however, no users found them abrasive or off putting. All three users stressed the need for the app to be personalizable. The following comments were made:

Hmmmm the colors are a bit dull, but then again, I’m not using this as a game or anything. I don’t mind it and the leaf design wouldn't stand out on my homescreen as a medical/mental health app.

ID1

It could be good to personalize this. Like, have a profile, that you can create a character for. Change some settings like the colors, or something.

ID3

The patients were less concerned about data protection and privacy than were the therapists.

I don't really think about the T’s & C’s to be honest. You get them on all apps now. They all need you to agree or you can’t use them. So I just scroll and accept.

ID2

All expatients were positive about the app as an addition to the paper processes in sessions, as this allowed for convenient tracking and reporting in the moment and sharing with their therapist. The following comments were made:

I think it’s a great idea. I wish I’d had it for my sessions. The paper was a pain when I was out and about. I could use this on the bus or if I was in a meeting and I felt myself slipping into a bad pattern of behavior. It would be better to make a note of that as it happened rather than at the end of the day.

ID2

Sometimes I forget what's happened in the week. I could use this to show my therapist in the session, like an overview.

ID3

Based on the user testing, the following five key user requirements were identified for the next iteration of the app that will be advanced for feasibility testing (will be reported in a follow-on paper) and assessment against UK National Health Services Digital Assessment Questions:

1. An early page is required for General Data Protection Regulations (GDPR) compliance, so that clients understand how their data will be processed and used, and can provide explicit consent for the therapist to have access to the data and the use of anonymized data for research purposes.

2. The whole client list will be hidden when one client is selected (on the therapist’s system).

3. There should be a place for the target problem procedure to be included, and the flow should be my target problem and my target problem procedure (actually called the target pattern description), followed by recognition rating and revision rating.

4. The mood scale (although disliked in terms of wording) needs to remain as presented owing to being a validated tool in CAT therapy and of added value for research purposes.

5. The “to do list” should be changed to “my exit list.”

## Discussion

### Principal Findings

We demonstrated that it is possible to design a relational awareness support tool for CAT clients that can be accessed on mobile phones and tablets. There was support for the development of the app from 50 CAT therapists who completed the online questionnaire. The results from the questionnaire showed that digital adoption in CAT practice is in its infancy and that any tool created would, rightly so, need to adhere to strict standards in GDPR. The therapist user testing also highlighted the need for strict GDPR in order to secure buy in and adoption from therapists and their host organizations. It is essential for any therapy support app to meet high data security standards, although expatients were less concerned about data security than therapists. This may have been the result of them being frequent users of a range of mobile apps and younger in age than the therapists; therefore, they had experience of accepting the terms and conditions of apps related to data sharing and storage. Conversely, they may simply not be aware of the potential for breaches in privacy with sensitive information. Assessing the final release version of the app against the UK National Health Service Digital Assessment Questions will ensure that all data security and regulatory issues are addressed. Three expatients of CAT found some functionality issues with the app and provided useful feedback that will improve the intuitiveness of the tool.

### Limitations

This early design work to develop a prototype app has been conducted using a medium-sized survey and a small cohort of mostly proficient users of technology for user testing. The results may not necessarily be replicated in a larger more diverse sample. However, for the purpose of designing an initial prototype for acceptability, adherence, and feasibility testing and for having a tool to assess against the National Health Service Digital Assessment Questions, the sample allowed us to develop content and highlighted key functionality issues for refinement of the app. This sample size is not uncommon in early prototyping research. Jakob Neilson is a world leader in the field of web and mobile app usability. His studies have shown that usability testing can be effectively carried out with a sample size as low as five [28,29]. This is because of the inverse exponential relationship between problems found and number of testers. Beyond five users, little new insight is gained, with 15 suggested as the number to remove 100% of usability issues. With a simple input app, such as the CAT-App, a sample size of six was deemed adequate for the identification of usability issues.

A simple yet effective user test is often overlooked when digital apps are developed, but is an essential element in creating mobile apps in mental health that meet global standards [10]. Owing to resource and time restraints, we were unable to explore and address all suggestions from the user testing, in particular, the value of an interactive map or more creative approaches to engaging patients in therapy. In this early work, we were keen to ensure that content did not interfere with fidelity to an evidence-based clinical intervention. Additional development and adaptation are things we will look to explore in future development of the app.

### Comparisons With Other Studies

The app enables the personal narrative and sequential diagrammatic reformulation of the patient to be preloaded and therefore enables each patient to be aware of his/her unique relational issues and the factors driving such issues. This is quite different from some of the more generic CBT apps. This creates new opportunities for clients to engage more fully in the self-awareness tasks of their therapy, without the inconvenience of referring back to paper diagrams, letters, and rating sheets. There is evidence that change in CAT is built on this foundation of self-awareness [9]. Therefore, the CAT-App aims to enable the patient to build an “observing self” and, from this position, change relational responding. These real-time self-awareness tasks can then be analyzed during therapy sessions by the client opening the app. The act of self-monitoring itself fosters important skills in reflective function. The patient-owned record also saves therapists’ time in maintaining records of their patients’ awareness and progress with changing problematic relationship patterns. This also saves in-session time as the client has already rated (possibly on numerous occasions out of the session, as opposed to just once in the session) the effectiveness of recognition and revision efforts. The graphical longitudinal feedback also enables clients to take more of a metaperspective on their progress with target problems and target problem procedures.

The CAT-App connects with and informs CAT treatment sessions, rather than being a stand-alone tool (as is the case with many CBT apps). The tool was also designed to map onto the phases of therapy, and it is therefore grounded in theory. The CAT-App is not a form of treatment, but rather a scaffold for effective treatment engagement in the relational recognition component of the approach in either 1:1 or group CAT therapy. This type of blended therapy is distinct from online or e-therapy, where the therapist and client communicate solely via messaging, video link, email, or phone. Blending face-to-face therapy with various technology-based interventions is now very common, but the mix varies. It ranges from minimal therapist support when following an online program to online or email support during primarily face-to-face therapy. The development of an app for mobile use specifically designed as an adjunctive tool to enhance a particular therapeutic method is innovative, although web-based programs have been developed, for example, in acceptance and commitment therapy [30]. Patients are prone to drop out of therapy when it is not meeting their needs, and this is, like deterioration in mental health during therapy, rightly seen as a negative outcome and one where therapists have a major ameliorating impact [31]. The app has potential for reducing drop out via an increase in engagement, particularly in the middle phase of treatment.

### Conclusions

Despite its technical complexity, patients found the CAT-App easy to use and therapists found it easy to configure. The data collected, when stripped of all personal identifiers, have great potential for research. The app removes the practical difficulties of releasing therapists and funding researchers to prepare and submit patient data, which are ubiquitous in practice-based research. Once informed consent has been obtained through the app, data collection and transfer are automated.

By introducing an adjunctive tool, there is a possibility that a well-tested therapy with known outcomes can be altered in ways that have unintended consequences, such as a negative impact on therapeutic alliance. In designing the app with therapists and former CAT clients, we sought to minimize these risks. Follow-on acceptability, adherence, and feasibility testing has been conducted, and the findings have been submitted for publication. Additional accessibility issues identified by therapists, including the need to make the app appropriate for patients with learning differences and visual impairment, will be addressed once funding is secured. Further research is clearly needed to test the impact of the app on clinical outcomes and will establish the best way to blend the app with CAT practice and specify the therapist competences required when using it.

## Abbreviations

CAT: cognitive analytic therapy
CBT: cognitive-behavioral therapy
GDPR: General Data Protection Regulations
PIN: personal identification number
